# Whole-body exposures to radiofrequency-electromagnetic energy can cause DNA damage in mouse spermatozoa via an oxidative mechanism

**DOI:** 10.1038/s41598-019-53983-9

**Published:** 2019-11-25

**Authors:** Brendan J. Houston, Brett Nixon, Kristen E. McEwan, Jacinta H. Martin, Bruce V. King, R. John Aitken, Geoffry N. De Iuliis

**Affiliations:** 1 Priority Research Centre for Reproductive Science, School of Environmental and Life Sciences, Discipline of Biological Sciences, University of Newcastle, Callaghan, NSW 2308 Australia; 2 School of Biological Sciences, Faculty of Science, Monash University, Clayton, VIC 3800 Australia; 3 School of Mathematical and Physical Sciences, University of Newcastle, Callaghan, NSW 2308 Australia; 4 Hunter Medical Research Institute, Cancer Research Program, New Lambton Heights, NSW 2305 Australia

**Keywords:** Mechanism of action, Mechanisms of disease, Spermatogenesis

## Abstract

Artificially generated radiofrequency-electromagnetic energy (RF-EME) is now ubiquitous in our environment owing to the utilization of mobile phone and Wi-Fi based communication devices. While several studies have revealed that RF-EME is capable of eliciting biological stress, particularly in the context of the male reproductive system, the mechanistic basis of this biophysical interaction remains largely unresolved. To extend these studies, here we exposed unrestrained male mice to RF-EME generated via a dedicated waveguide (905 MHz, 2.2 W/kg) for 12 h per day for a period of 1, 3 or 5 weeks. The testes of exposed mice exhibited no evidence of gross histological change or elevated stress, irrespective of the RF-EME exposure regimen. By contrast, 5 weeks of RF-EME exposure adversely impacted the vitality and motility profiles of mature epididymal spermatozoa. These spermatozoa also experienced increased mitochondrial generation of reactive oxygen species after 1 week of exposure, with elevated DNA oxidation and fragmentation across all exposure periods. Notwithstanding these lesions, RF-EME exposure did not impair the fertilization competence of spermatozoa nor their ability to support early embryonic development. This study supports the utility of male germ cells as sensitive tools with which to assess the biological impacts of whole-body RF-EME exposure.

## Introduction

With rapid advances in technology and increasing demand for electronic communication, mobile phone usage has become virtually ubiquitous in the developed world^1^. Mobile phone devices receive and emit radiofrequency-electromagnetic energy (RF-EME) to transfer information, and accordingly our exposure to this form of energy is now unprecedented. Thus there is a clear imperative to establish public safety guidelines around the use of these mobile devices. It is, however, difficult to meet this demand due to a current lack of understanding concerning how RF-EME interacts with biology. While to date, no overwhelming clinical effects have been associated with RF-EME exposure^2–6^, multiple studies suggest that this form of energy can elicit subtle detrimental effects on biological systems^7–10^. Accordingly, the International Agency for Research on Cancer have yet to dismiss the risks of RF-EME, instead classifying this form of energy as a potential carcinogen. While we continue to debate the biological effects of chronic RF-EME exposure, a growing body of evidence now proposes that acute *in vitro* RF-EME exposure can elicit oxidative stress in a range of model cell lines^7,9,11–13^. A leading hypothesis to account for the mechanistic basis of this response is that RF-EME targets the mitochondria, leading to perturbation of proton flux across the inner mitochondrial membrane and promoting electron leakage from the electron transport chain. The resultant formation of superoxide anion serves as a progenitor for additional reactive oxygen species generation (ROS), eventually creating a ROS imbalance and a state of oxidative stress^1,12^.

The potential for this mechanism of biophysical interaction provides the impetus for well-designed studies to ascertain the effect of RF-EME following whole-body irradiation regimens that more accurately mimic human exposure. In this context, a focus on the male reproductive system is justified owing to the common practice of storing mobile phone devices in the pant pocket, placing them in close proximity to the reproductive tract. Further emphasizing the relevance of the male reproductive system is mounting evidence that male germ cells are particularly susceptible to RF-EME^14^ and the associated production of oxidative stress^7,12^. Indeed, it has been shown that spermatozoa provide a sensitive model to study the specific physical and chemical responses to RF-EME^15^. The situation arises because of the unique architecture and metabolism of spermatozoa, which places these cells at heightened vulnerability to damage by free radicals^16^. Moreover, spermatozoa provide a readily assessable means of monitoring adverse biological effects, through functional parameters such as motility, or more detailed analysis that can pinpoint biochemical disruption and more subtle endpoints such as the accumulation of DNA damage. Besides serving as a sensitive model, these cells are also clinically important, since the induction of DNA damage in the male germ line contributes to infertility^16^ and has the potential to propagate in the embryo, altering developmental trajectory and the health of the offspring^16,17^.

**Figure 1:**
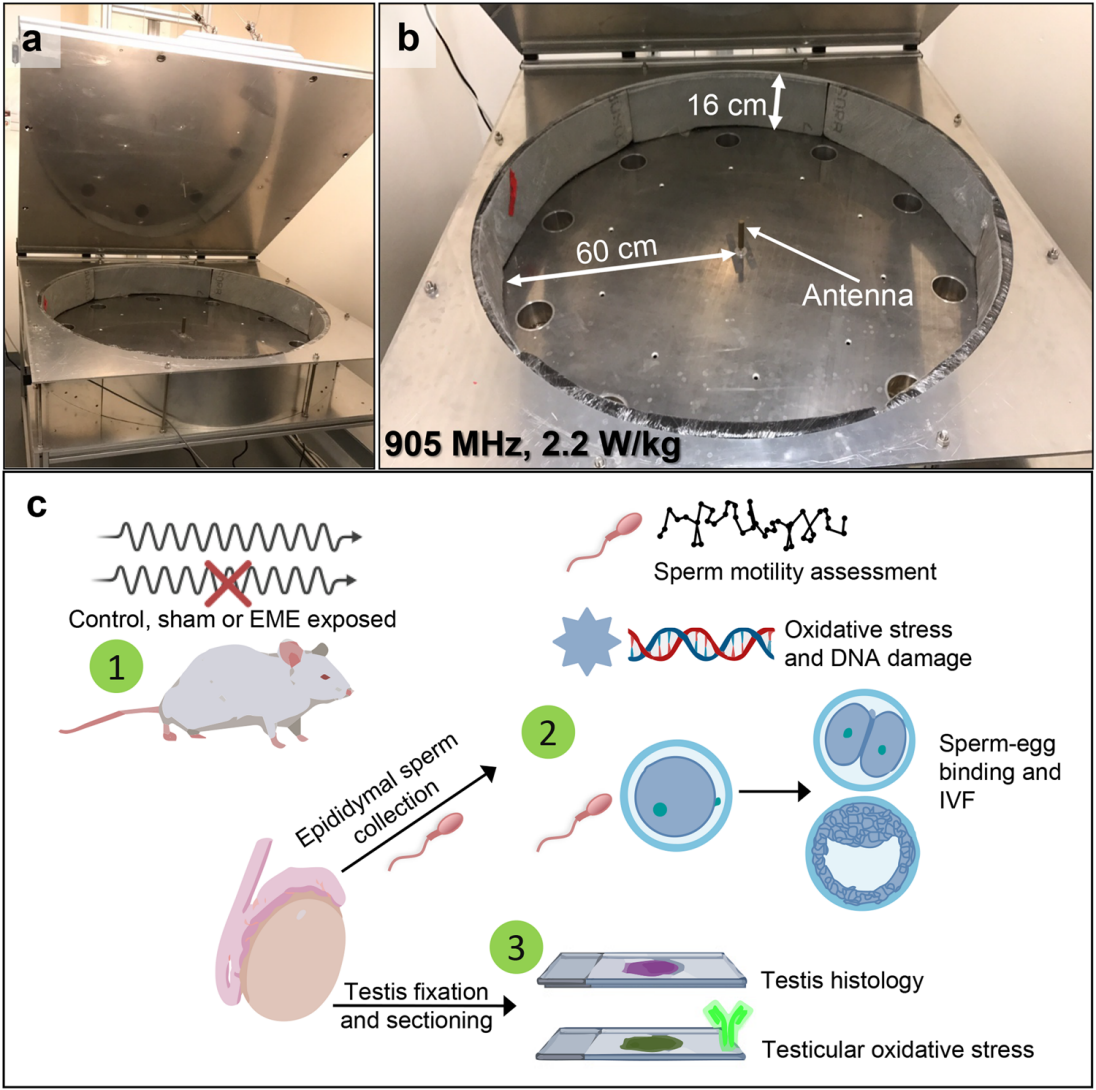
Waveguide instrument used to deliver whole-body RF-EME exposure. Shown are (**a**) the complete waveguide apparatus with lid in open configuration and (**b**) close-up view illustrating the dimensions of the inner chamber. (**c**) A graphical experimental overview. (1) Mice were RF-EME or sham exposed for 1, 3 or 5 weeks and compared to a control population that did not enter the apparatus (untreated). Mice were culled and their spermatozoa were examined using sperm functional assays and a variety of oxidative stress assays (2). The testes of these mice were also examined for gross histological abnormalities and for markers of oxidative stress, via tissue sections (3).

To date, a handful of studies have sought to assess the effects of RF-EME on the male germ line. However, the majority of these studies have focused on isolated spermatozoa or immature male germ cells^12,15,18–21^. While this approach is conducive to examination of the intricate biochemical and cellular responses to direct RF-EME exposure, the use of alternate *in vivo* rodent models is likely to present a closer clinical representation of exposure, which can also serve to extend our understanding of EME-perturbed biochemical pathways highlighted from *in vitro* studies. Whole body models afford the added advantage that they enable observation of the holistic effects of RF-EME on all stages of male germ cell development^22^, encompassing the differentiation of germ into spermatozoa and their subsequent functional maturation as they transit the epididymis. With a sustained interest in establishing the biophysical mechanism(s) of action for RF-EME on biology, we report the use of a mouse model to probe reproductive stress following whole-body RF-EME exposure regimens. Specifically, a dedicated waveguide machine (Fig. 1), similar to that developed by Puranen and colleagues^23^, was constructed to facilitate exposure of unrestrained mice to RF-EME at 905 MHz with a specific absorption rate (SAR) of 2.2 W/kg. Mice were exposed to RF-EME for 12 h per day, over a period of between 1 to 5 weeks and subsequently the testes and epididymides were collected to investigate the effects of RF-EME on spermatogenesis and sperm function.

## Results

### Whole-body RF-EME exposure does not elicit gross histological changes in the mouse testis

**Figure 2:**
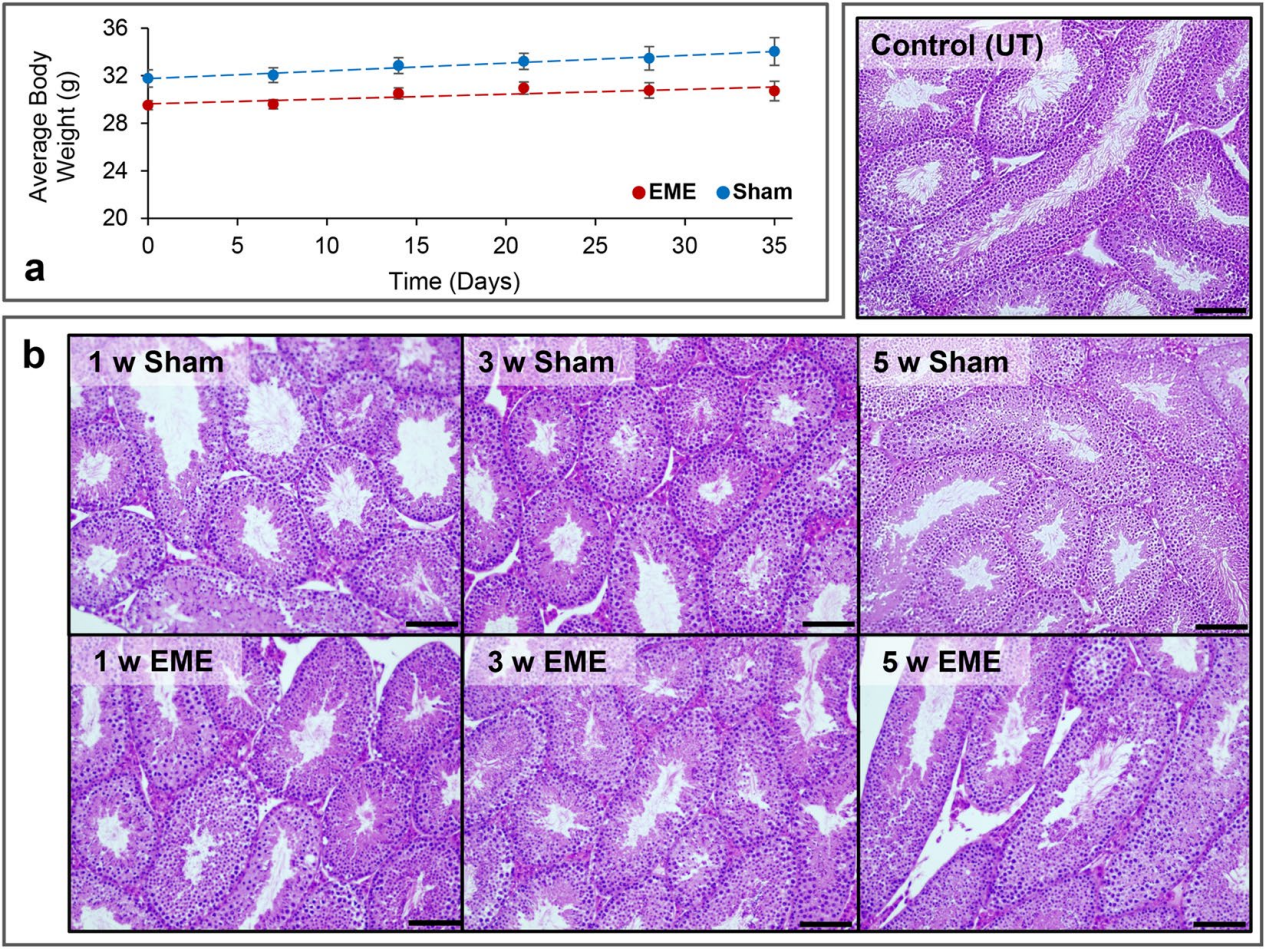
The effect of RF-EME on the growth and testis morphology of C57BL/6 mice. (**a**) Mice were weighed at weekly intervals to investigate the effects of RF-EME on body mass against sham exposed males (n = 8–20 mice measured/treatment group). Red circles represent the mean weight of EME treated mice, whereas blue circles represent the sham exposed group (**b**) Haematoxylin and eosin staining of testis sections was conducted to facilitate comparison of gross seminiferous tubule morphology (n = 3 mice/treatment group). Scale bar represents 400 µm.

Following exposure of unrestrained mice to whole-body RF-EME exposure, we first examined the effects of our varied regimens on the average growth rate (Fig. 2a) of irradiated animals over the 5 weeks; revealing no changes in rate between the sham and RF-EME exposure groups. Similarly, gross testis morphology of sham and RF-EME exposed mice also remained comparable to that of control mice (Fig. 2b), with all samples exhibiting healthy tubule growth and extensive germ cell proliferation irrespective of the duration of exposure. All mice were 8 weeks of age at the commencement of the 1, 3 and 5 week study, however, some variance in body weight between cohorts was observed on their arrival. Nevertheless, no significant change in average growth rate was recorded between exposures, over the 35-day study (Fig. 2a).

**Figure 3:**
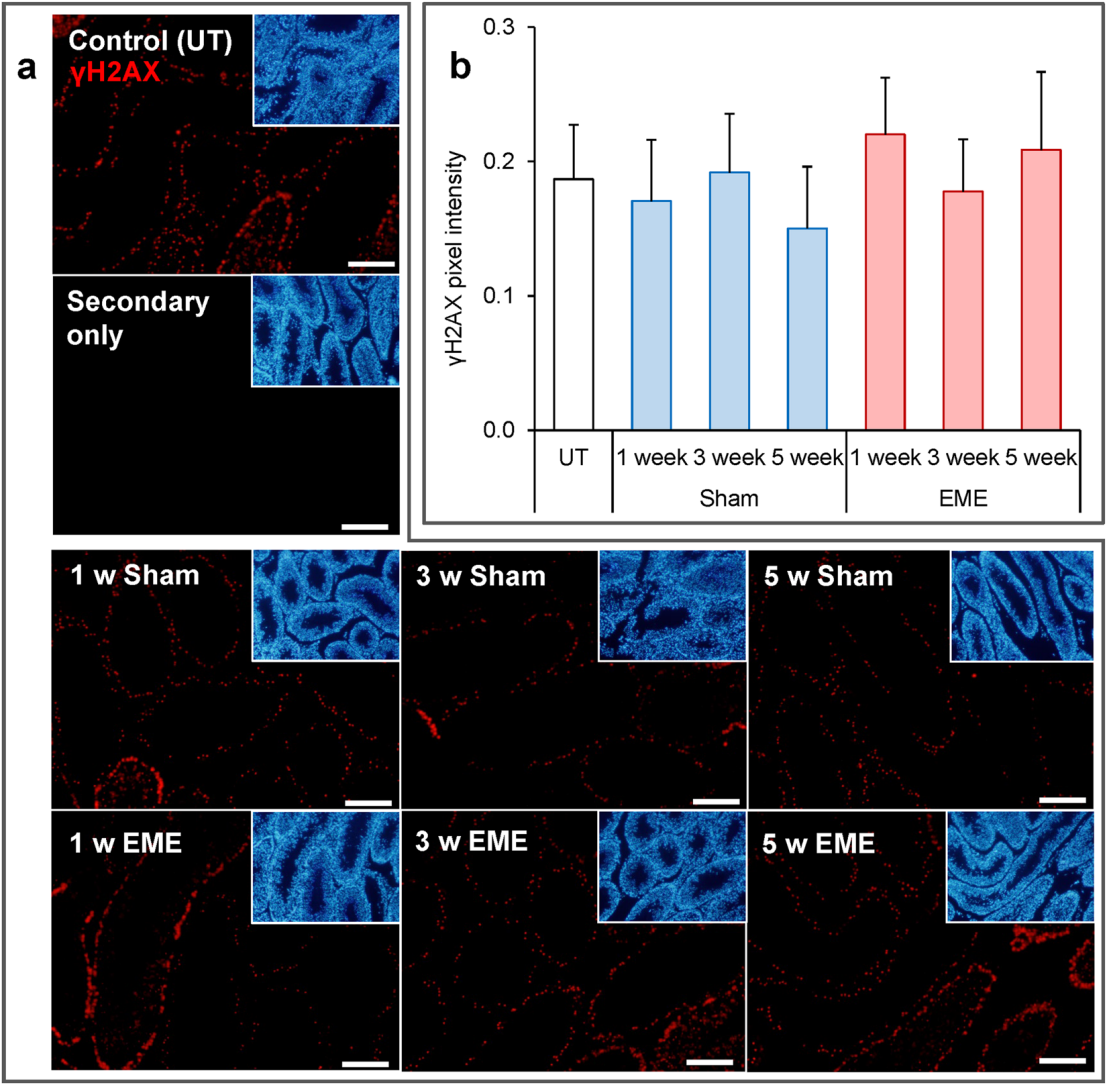
RF-EME exposure does not induce γH2AX expression in the testis. Testis sections from untreated control animals (UT), as well as those of the sham and RF-EME exposure groups, were probed with anti-phospho-γH2AX antibodies (red) to detect DNA double strand breaks. (**a**) Representative images are depicted, with scale bar equating to 400 µm. A secondary antibody only control is also included. Corresponding DAPI (blue) stained images, illustrating tubule morphology are included as insets included in the upper right corner of each panel. (**b**) Analysis of pixel intensity was performed on the germ cell population within the seminiferous tubules in order to quantify γH2AX expression levels across treatments. Graphical data are presented as mean + SEM (n = 3 mice/treatment group, with 8–25 tubules being analyzed for each testis).

**Figure 4:**
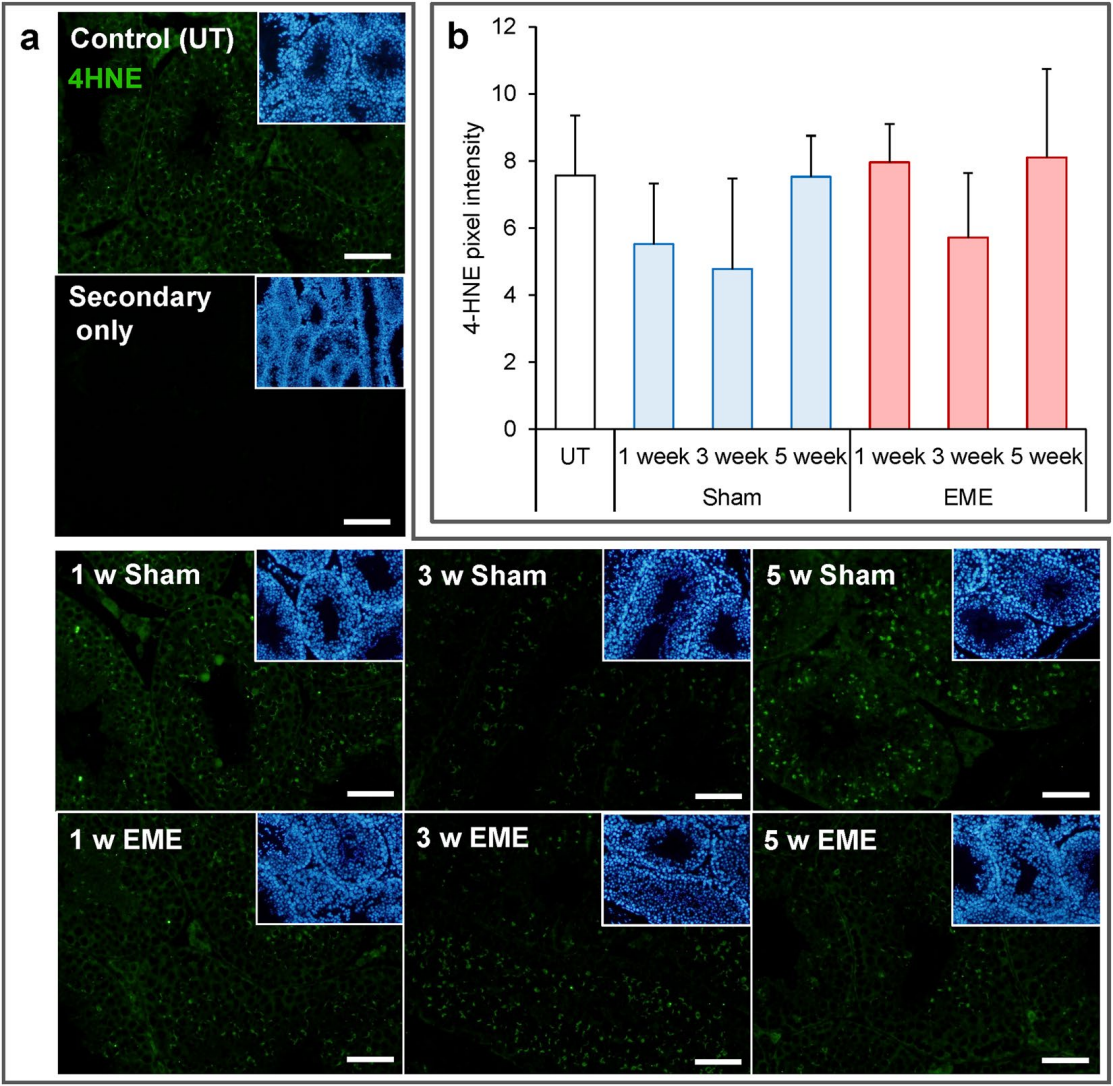
RF-EME exposure does not induce elevated 4-hydroxynonenal formation in the testis. Testis sections from untreated control animals (UT), as well as those of the sham and RF-EME exposure groups, were probed with anti-4-hydroxynonenal antibodies (green) to detect by-products of lipid peroxidation. (**a**) Representative images are depicted, with the scale bar equating to 200 µm. A secondary antibody only control is also included. Corresponding DAPI stained images illustrating tubule morphology are included as insets included in the upper right corner of each panel. (**b**) Analysis of pixel intensity was performed on the germ cell population within the seminiferous tubules in order to quantify 4-hydroxynonenal expression levels across treatments. Graphical data are presented as mean + SEM (n = 3 mice/treatment group, with 10–20 tubules being analyzed for each testis).

Guided by our previous studies in which we have shown that *in vitro* RF-EME exposure can induce a state of oxidative stress, leading to DNA damage in some male germ cell types^7,12^, we next explored the levels of DNA fragmentation and lipid peroxidation present within the testes of RF-EME exposed animals. For the former analysis, testis sections were probed with an anti-γH2AX antibody, a marker of DNA double strand breaks (Fig. 3). This revealed modest levels of DNA damage, which was largely restricted to meiotic germ cells within the seminiferous tubules. Furthermore, this tissue localization and levels of γH2AX staining were consistent across the panel, with no effect observed due to EME exposure (*p* = 0.07) or time. With regard to lipid peroxidation (Fig. 4), we documented a similar response, with no substantive increases in the lipid peroxidation product, 4-hydroxynonenal being detected within the testis sections of any RF-EME treatment group with respect to the untreated or sham controls (*p* = 0.22).

### Whole-body RF-EME exposure adversely impacts the vitality and motility profiles of mature spermatozoa

**Figure 5:**
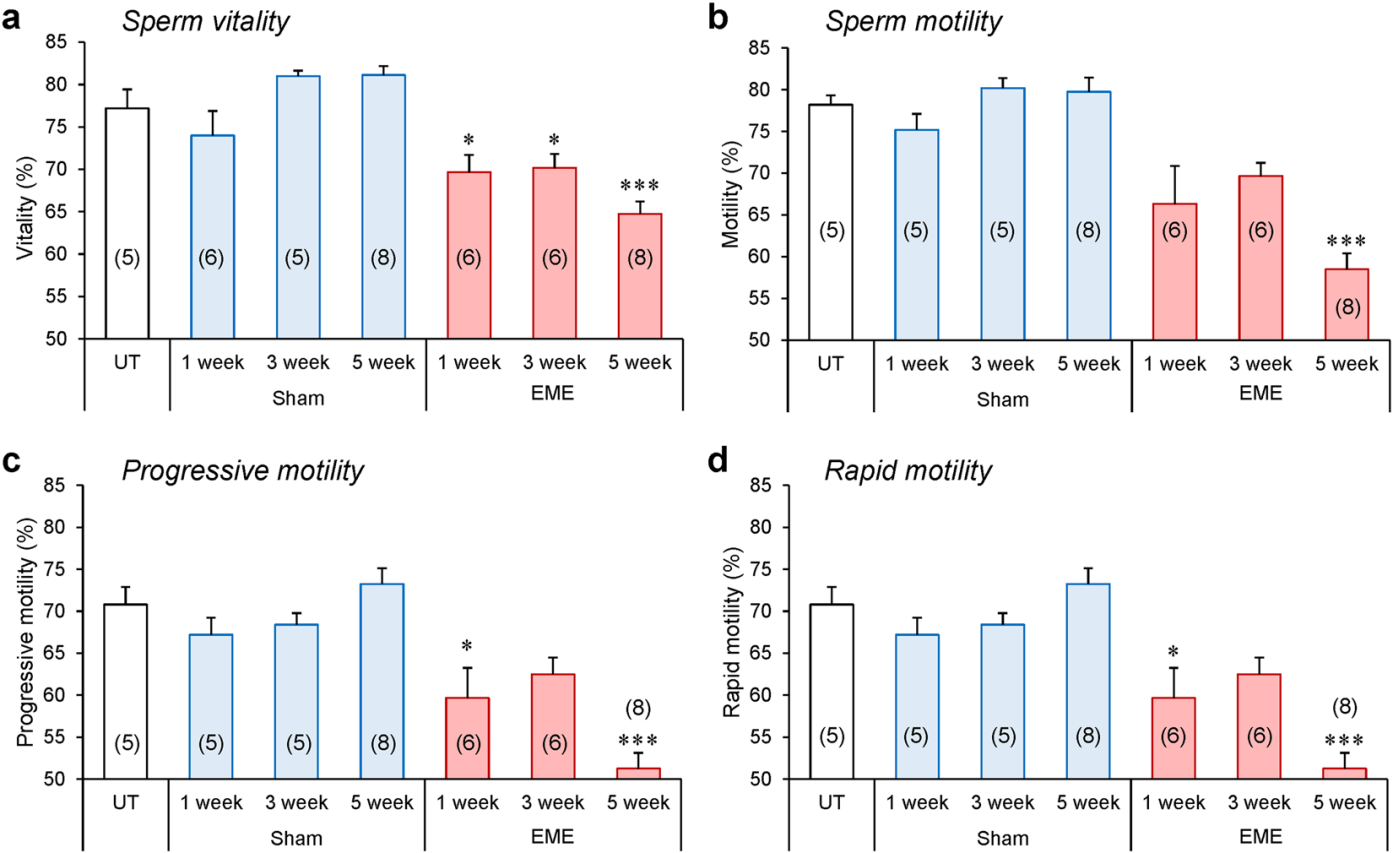
Sperm vitality and motility declines in response to RF-EME exposure. Spermatozoa were collected from the cauda epididymis of untreated control animals (UT), as well as those of the sham and RF-EME exposure groups. (**a**) Sperm vitality was assessed via the eosin-exclusion method. Next, the percentage of sperm displaying (**b**) any form of motility, (**c**) progressive motility, and (**d**) rapid motility was determined using computer assisted semen analysis. Data are presented as mean + SEM (n = 5–8 mice/treatment group), with a minimum of 100 spermatozoa being analyzed from each animal). The number of biological replicates used is denoted in each bar. *P < 0.05, ***P < 0.001.

To explore the effect of *in vivo* RF-EME exposure on mature spermatozoa, we next investigated the outcomes of our irradiation regimen on sperm motility and vitality (Fig. 5). It was observed that the total number of live spermatozoa isolated from the cauda epididymis was diminished with RF-EME exposure (*p* < 0.05) (Fig. 5a), an effect that was particularly evident after 5 weeks of exposure (*p* < 0.001); whereas no changes were observed in our sham-exposed populations. In a similar manner, we noted a significant reduction in the percentage of motile spermatozoa isolated from RF-EME exposed mice following a treatment regimen extending over 5 weeks (*p* < 0.05) (Fig. 5b). This reduction in overall sperm motility occurred commensurate with defects in the objective measurements of progressive and rapid sperm motility (Fig. 5c,d) in exposed mice. In this regard, the impact on both parameters was again most notable following 5 weeks of exposure (*p* < 0.001). Conversely, spermatozoa isolated from the sham exposure groups displayed no such changes in their vitality or motility profile; with both parameters remaining indistinguishable from those documented in an unexposed control group of males.

### Whole-body RF-EME exposure elevates oxidative stress and DNA damage in mature spermatozoa

**Figure 6:**
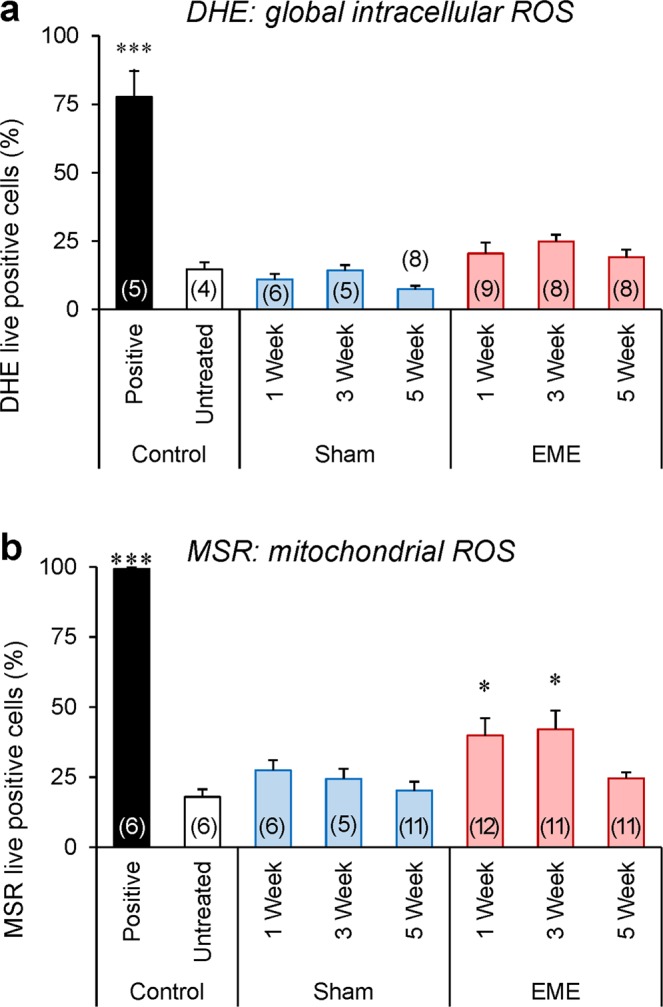
Exposure to RF-EME stimulates the generation of mitochondrial reactive oxygen species. Spermatozoa were isolated from the cauda epididymis of untreated control animals, as well as those of the sham and RF-EME exposure groups. These cells were pre-loaded with fluorescent probes and then analyzed using flow cytometry to assess their generation of reactive oxygen species (ROS). (**a**) Global levels of ROS generated in the sperm cell was assessed with the dihydroethidium (DHE) probe. (**b**) Alternatively, mitochondrial ROS generation was investigated with the MitoSOX Red (MSR) probe. In both instances, a minimum of 10,000 spermatozoa were assessed from 5–12 of animals and data are presented as mean + SEM. The number of biological replicates used is denoted in each bar. *P < 0.05.

To determine whether the functional lesions in motility and vitality documented in the spermatozoa of RF-EME exposed mice were linked to the induction of oxidative stress, we next investigated the levels of cellular and mitochondrial ROS present in these cells (Fig. 6). Specifically, the dihydroethidium (DHE) fluorescent probe was utilized to provide insight into levels of cellular ROS production (Fig. 6a). Approximately 14% and 75% spermatozoa stained positively for DHE in the negative (untreated) and positive (i.e. hydrogen peroxide exposed) control populations, respectively. When the experimental groups were analyzed, neither the sham nor the RF-EME treatment conditions resulted in a significant deviation from basal ROS generation detected by DHE labeling. This was in contrast to mitochondrial ROS production, where the MitoSOX Red (MSR) probe (Fig. 6b) revealed a significant, two-fold elevation in ROS generation within the sperm mitochondria of animals exposed to RF-EME for periods of either 1 or 3 weeks, compared to the control and sham-exposed cell populations (*p* < 0.05). Intriguingly, sperm mitochondrial ROS generation had normalized to basal, control levels following 5 weeks of RF-EME exposure.

**Figure 7:**
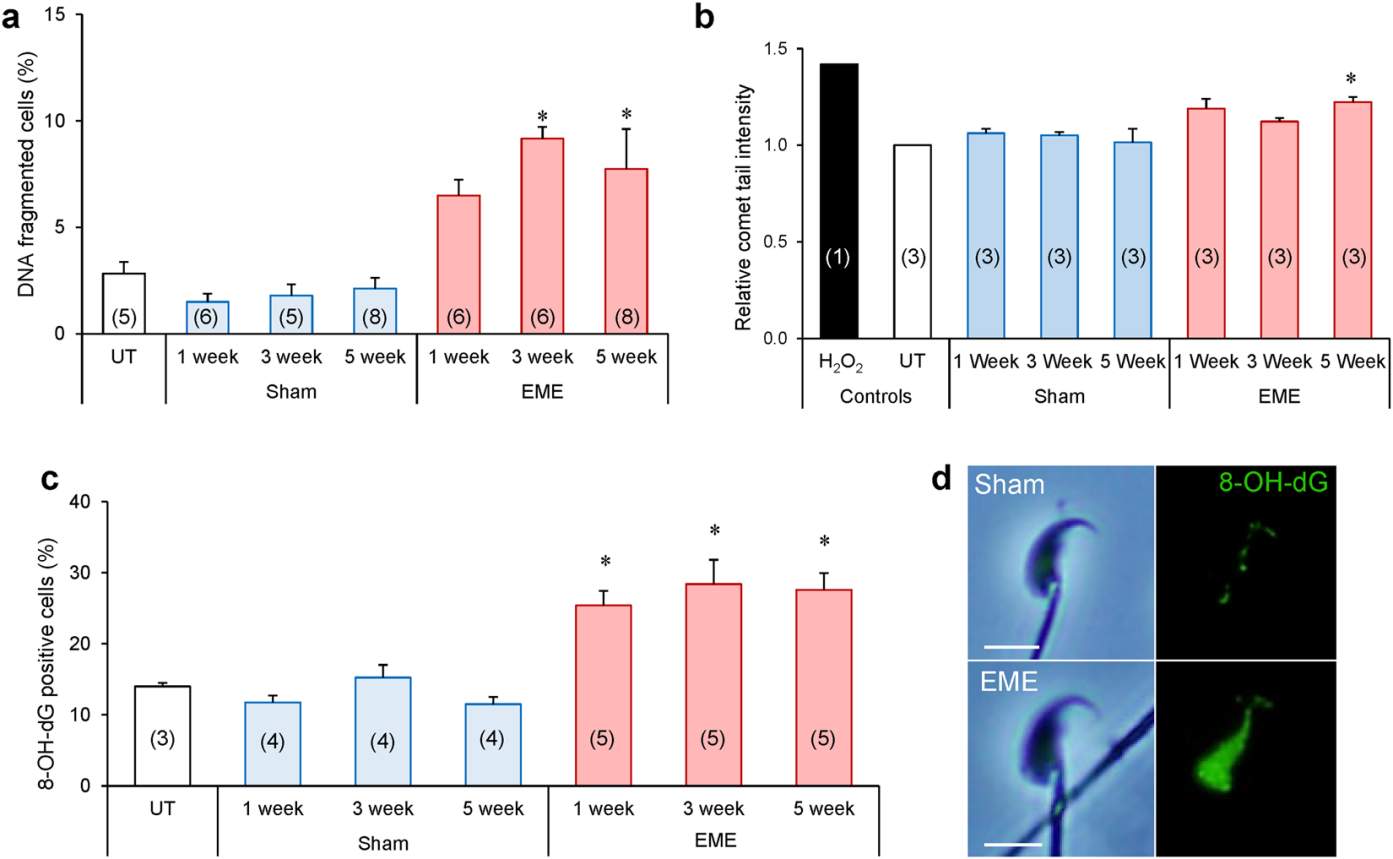
RF-EME exposure induces oxidative DNA damage in spermatozoa. Spermatozoa were isolated from the cauda epididymis of untreated control animals (UT), as well as those of the sham and RF-EME exposure groups. These cells were assessed for DNA fragmentation using (**a**) the halo assay showing the percentage of cells fragmented (n = 5–8 mice/treatment group, each with 100 sperm assessed for each replicate) and then (**b**) quantified by the alkaline comet assay, expressed as percentage tail intensity and normalized to control data for each run (n = 3 mice/treatment, each with 50–70 sperm cells assessed). (**c**) To extend this DNA integrity analysis, sperm were evaluated for oxidative DNA adducts via labelling with anti-8-hydroxy-2-deoxyguanosine (8-OH-dG) antibodies (n = 3–5 mice/treatment). (**d**) Representative images of spermatozoa stained with the 8-OH-dG antibody from the 5 week sham and RF-EME exposed populations are included. The number of biological replicates used is denoted in each bar. Data are presented as mean + SEM. *P < 0.05, **P < 0.01.

DNA damage assays were next employed to gain insight into the consequences of RF-EME induced ROS generation on the DNA integrity of mouse spermatozoa (Fig. 7). The halo assay (Fig. 7a), which evaluates DNA integrity based on the presence or absence of a halo-like stained DNA structure, revealed a modest but significant increase (i.e. ~5–6%) in the percentage of DNA-fragmented spermatozoa following 3 and 5 weeks of RF-EME exposure (*p* < 0.05). Consistent with these findings, the application of an alkaline comet assay (Fig. 7b) confirmed that whole-body RF-EME exposure stimulated sperm DNA fragmentation. After 1 week, sperm DNA fragmentation was elevated by 18%, however, this increase only gained significance after 5 weeks of exposure (23% increase in fragmentation; *p* < 0.05). Given the elevation in mitochondrial ROS, we next demonstrated that this DNA damage was likely oxidative in nature, highlighted by an increase in the percentage of RF-EME exposed sperm displaying positive staining for 8-hydroxy-2-deoxyguanosine (8-OH-dG; Fig. 7c); a biomarker of oxidative DNA damage. Indeed, across each of the three exposure times assessed, RF-EME induced a significant (*p* < 0.05) increase in 8-OH-dG labelling relative to control and sham exposed populations. As anticipated, 8-OH-dG labelling was localized to the nuclear compartment of the sperm head and was consistently more intense in RF-EME treated spermatozoa (Fig. 7d).

### Whole-body RF-EME exposure does not impair the fertilization competence of spermatozoa

**Figure 8:**
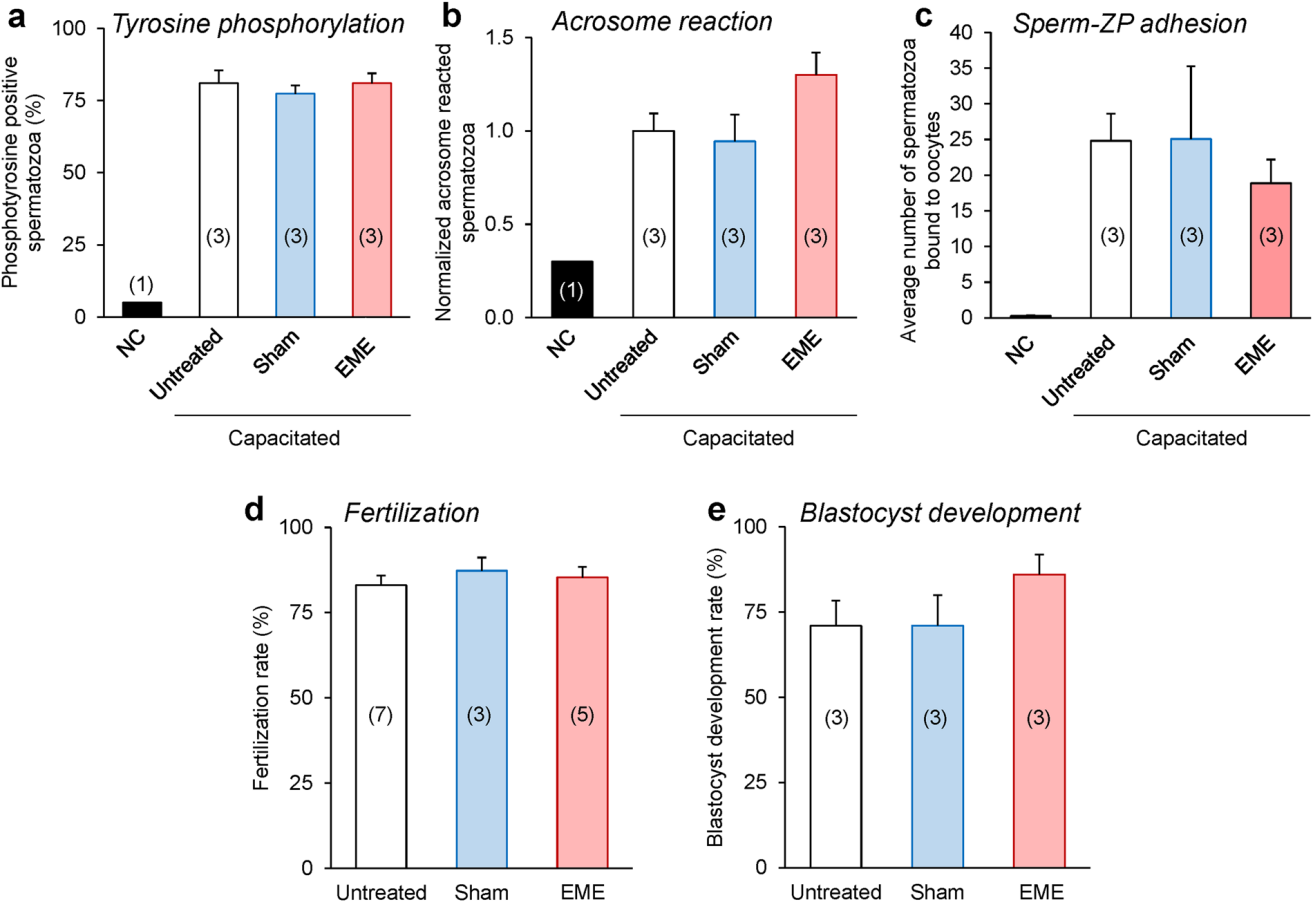
RF-EME exposure did not compromise the fertilization competence of spermatozoa. Spermatozoa were isolated from the cauda epididymis of untreated control animals, as well as those of the 5-week sham and RF-EME exposure groups. These cells were driven to capacitate and then assessed for (**a**) anti-phosphotyrosine labeling of the sperm flagellum, and (**b**) their ability to undergo a calcium ionophore induced acrosome reaction [assessed via peanut agglutinin (PNA) labeling of the sperm outer acrosomal membrane with values being normalized to the untreated control], and (**c**) binding to the zona-pellucida (ZP) of homologous oocytes (the average number of spermatozoa bound to ZP intact oocytes is shown). In each instance a non-capacitated (NC) population of spermatozoa from untreated animals was included as a negative control. Alternatively, spermatozoa were examined for their ability to (**d**) fertilize oocytes *in vitro* and subsequently (**e**) support early embryo development through to the blastocyst stage. In all instances, assessed spermatozoa were isolated from each of three animals and data are presented as mean + SEM, except for (**d**), where 3–7 mice were used. The number of biological replicates is shown in each bar. (**a, b**) A minimum of 100 spermatozoa from each animal were assessed for phosphotyrosine labelling of the sperm flagellum, and PNA labelling of the acrosome. (**c**, **d**) 8–10 oocytes per replicate were assessed for sperm-ZP binding and 11–30 for fertilization, and (**e**) 11–30 embryos were assessed for blastocyst development.

In order to determine if RF-EME mediated induction of sperm DNA damage was sufficient to compromise the fertilization competence of these cells, we undertook an assessment of selected markers of sperm capacitation and *in vitro* fertilization success utilizing the spermatozoa from 5 week RF-EME exposed mice (Fig. 8). Of the capacitation markers assessed, neither the number of sperm displaying complete flagellum phosphotyrosine labelling (Fig. 8a) or the ability to undergo a calcium ionophore induced acrosome reaction (Fig. 8a) differed significantly between the control and RF-EME treatment groups. Similarly, the average number of spermatozoa bound to the zona pellucida of fixed oocytes was also unchanged across our control (25), sham (25) and RF-EME exposed (19) populations (Fig. 8c,d; *p* = 0.99). As an extension of this assessment of sperm function, the ability of spermatozoa from all three treatment groups to achieve fertilization and progression to the blastocyst stage of development was then investigated. Exposure to RF-EME under our regime, did not exert any observable effect on fertilization rate (Fig. 8d), with all treatment groups resulting in the fertilization of between 83–87% of inseminated oocytes. Furthermore, when these zygotes were cultured through to the blastocyst stage of development (Fig. 8e), a modest increase was observed in the development rate of the RF-EME group, although this did not prove to be significantly different from the sham exposed or untreated sperm groups.

## Discussion

Several lines of evidence now propose RF-EME to be capable of inducing a state of oxidative stress in a variety of cell types^24–27^, including the male germline^7,12^. It is also well established that spermatozoa are particularly sensitive to oxidative insults, a phenomenon that may be traced to their surplus of oxidizable substrates and restricted antioxidant capacity^16,28^. What remains less certain is how RF-EME is capable of inducing such cellular responses in the absence of a thermal induction mechanism. In seeking to resolve this question, here we have utilized an *in vivo* exposure model that not only approximates the complexities of environmental RF-EME exposures, but also enables the dissection of RF-EME effects on key stages of male germ line development. Specifically, our exposure regimen enabled determination of the interaction of RF-EME with spermatozoa held exclusively within the luminal environment of the epididymis (1 week exposure), as well as those exposed during their progression through a spermatogenic cycle and transit of the epididymal tract (5 week exposure). Consistent with our previous *in vitro* investigations^7,12^, we here contribute data to support the dysregulation of sperm mitochondria as a pivotal target for driving RF-EME associated stresses in the male reproductive system.

In contrast to previous reports of disorganized testicular architecture and spermatogenesis arising from whole-body RF-EME exposure^29,30^, the supraphysiological treatment regimen implemented here did not support these findings, with no changes to gross testicular histology observed. Similarly, both the somatic and germline tissue within the seminiferous tubules also proved recalcitrant to RF-EME induction of DNA damage or lipid peroxidation. Such findings are not entirely unexpected given the lack of robust evidence to support the ability of environmental RF-EME exposure conditions to elicit such obvious overt tissue damage^1^. Rather, on the basis of prevailing evidence we consider that any biophysical RF-EME interactions would likely result in more subtle phenotypic changes^1^, thus justifying our primary focus on the male germ line as a sensitive model cell type^16^ to explore mechanisms of RF-EME mediated stress. Accordingly, we observed a clear attenuation of sperm motility, occurring in concert with increased mitochondrial ROS generation, after 1 and 3 weeks of whole-body RF-EME exposure. In the absence of commensurate increase in cytosolic ROS production, these data provide correlative evidence that sperm mitochondria are indeed prone to RF-EME dysregulation and that the ensuing production of ROS was sufficient to compromise the most vulnerable aspects of sperm cell function. Additional support for this model rests with a growing body of literature implicating RF-EME in the generation of a state of oxidative stress in a variety of cell types other than the male germ line^11,13,31–34^.

An interesting observation to arise from our study was that the induction of mitochondrial ROS generation after 1 and 3 weeks of RF-EME exposure was followed by an apparent normalization of mitochondrial ROS after an additional 2 weeks of exposure. At present, we remain uncertain what mechanism(s) could account for the mitigation of this response, but speculate they may be associated with reduced mitochondrial function in germ cells subjected to prolonged RF-EME exposure, or that these cells are capable of responding to this challenge through an elevation of intrinsic antioxidant defenses. As an extension of this hypothesis, it is possible that the male reproductive tract also mounts a protective response to chronic RF-EME via an upregulation of exogenous antioxidant production. In keeping with this notion, it has been shown that the concentrations of both vitamin A and E increase in the testis of RF-EME exposed rats^35^. Alternatively, this phenomenon could be linked to morphological changes in the mitochondrion during spermatogenesis^36^; such as the extensive vacuolization these organelles undergo during the maturation of spermatogonia to spermatocytes^37^. Accompanying such changes, mitochondrial activity is also elevated in spermatocyte and spermatid populations, whereas mature mouse spermatozoa are known to limit their investment into oxidative phosphorylation and instead utilize glycolysis to meet their energy demands^38^. Finally, there is also evidence that the mitochondria of caput epididymal spermatozoa are silenced^39^, which may afford some protection against perturbed mitochondrial ROS production while also identifying a dynamic sensitivity of spermatozoa. The cauda epididymal spermatozoa sampled after enduring 5 weeks of EME exposure, will comprise a mixture of cells exposed during various stages of germ cell development and maturation, however the majority of the cells will likely have encountered EME as morphologically mature spermatozoa, which may house less vulnerable mitochondria. Irrespective of the mechanism(s) responsible for suppression of ROS production, the downstream detrimental legacy of RF-EME exposure persisted in mature spermatozoa after 5 weeks of treatment as evidenced by the demonstration that these cells suffered the highest losses of vitality and motility. Thus, although the production of mitochondrial ROS was ameliorated in spermatozoa after 5 weeks of RF-EME exposure, these cells were unable to repair the oxidative damage they sustained during prior exposure.

The identification of sperm motility as being vulnerable to RF-EME exposure is consistent, independent evidence that this functional attribute is among of the first to succumb to elevated levels of ROS^40,41^. ROS mediated lipid peroxidation is known to drive the production of reactive aldehydes, such as 4-hydroxynonenal, which causes irreversible protein modifications and alkylation of the sperm axoneme^42^. Where oxidative stress levels may spike at an earlier window in sperm development, limiting the amount of detectable ROS in the sperm collected at 5 weeks, these cells can retain hallmarks of this pathology, in the form of oxidized DNA lesions. Consistent with this notion, we detected an increase in the oxidative stress biomarker, 8-OH-dG, in the nuclei of sperm across all exposure regimens; indicating abundant guanosine oxidation and supporting RF-EME as a mediator of oxidative stress. A similar finding has been reported by Liu *et al*.^19^, who documented a significant elevation in 8-OH-dG formation in spermatocytes exposed to RF-EME. Accompanying oxidative DNA damage, we observed elevated sperm DNA fragmentation in the form of single strand breakage following whole-body RF-EME exposure. These data accord with the enhanced levels of DNA fragmentation documented in spermatozoa^7,21,43^ and spermatocytes^19^ exposed to RF-EME; a phenomenon that may describe a continuum of DNA damage, originating from oxidative DNA insults^44^. While further studies are required to pinpoint variations in the sensitivity of different germ cell populations to RF-EME *in vivo*, our data suggests that a window of vulnerability may extend across both testicular and post-testicular (i.e. epididymal) phases of development.

Not with standing an elevation in oxidative stress mediated DNA damage and an attendant reduction of motility, the spermatozoa recovered from mice exposed to 5 weeks of whole-body RF-EME did not display any associated lesions in their fertilization potential. Thus, these cells retained their ability to capacitate, acrosome react, and bind zona pellucidae at rates that were statistically indistinguishable from those of untreated and sham exposed mice. Moreover, these spermatozoa were capable of supporting normal rates of *in vitro* fertilization and early embryo development. In seeking to reconcile these data, a key limitation is that the *in vitro* fertilization strategy adopted in this study, introduced selection bias for the higher quality, motile spermatozoa which potentially harbour only basal levels of DNA damage. Even in the lowest motility group, after 5 weeks of EME exposure, 60% of the recovered cells remain motile. This notion is consistent with studies of human IVF patients, which have revealed that *in vitro* assays of sperm-zona pellucida binding are highly selective for spermatozoa with intact DNA and normal motility profiles^45^. Alternatively, it is possible that the burden of DNA damage harbored by the fertilizing spermatozoon was sufficiently resolved by the oocyte. In any case, this clearly illustrates, reassuringly, that even at the supraphysiological regimens of whole-body RF-EME exposure used in this study, no overt impairment to fertilization potential and early embryo development was observed. This is perhaps in alignment to the lack of overt morphological changes observed in the reproductive tissue of exposed mice, confirming observations that environmental RF-EME does not contribute to gross biological damages. In this context, and given the evidence of cellular oxidative impacts, we cannot yet discount the possibility of transmission of subtle phenotypic or epigenetic changes in the offspring. Thus, future studies focused on trans- and multi-generational outcomes will likely play a key role in resolving any potential for cumulative changes caused by RF-EME. While more targeted investigations into this aspect of exposure is warranted, it is perhaps comforting that whole-body chronic exposure (life-long, 24 h/day) to electromagnetic fields has been reported to elicit no harmful effects on the fertility or development of mice over four successive generations^46^.

In summary, our evidence supports the hypothesis that sustained whole-body RF-EME is capable of inducing a state of oxidative stress in the male germ line, a cell vulnerable to the effects of ROS. Furthermore, our data further implicate the mitochondria as the target for RF-EME biophysical interaction, with a consequential elevation of mitochondrial ROS generation being linked to reduced motility and elevated oxidative DNA damage and DNA fragmentation in the spermatozoa of exposed males. Whilst these lesions were not sufficient to compromise fertilization competence or early embryo development, it will nonetheless be of interest to investigate the transgenerational influence of whole-body RF-EME in future studies.

## Methods

### Chemical reagents

The reagents used in this study were purchased from Sigma-Aldrich (St. Louis, MO, USA) unless stated otherwise. Fluorescent probes were purchased from Thermo Fisher Scientific (Waltham, MA, USA), unless otherwise stated. All fluorescence imaging was performed using a Zeiss Axioplan 2 fluorescence microscope (Carl Zeiss MicroImaging GmbH, Jena, Germany).

### Waveguide design and whole-body RF-EME exposure regimens

Adult (>8 weeks) male C57BL/6 mice were irradiated with 2 W/kg and 905 MHz RF-EME in a waveguide (Fig. 1) for 12 h daily, during a night (7 pm–7 am) cycle while the waveguide lid was closed. This waveguide was constructed by the Physics Department at the UON and comprises a cylindrical aluminium chamber (radius of 60 cm and depth of 16 cm) and mechanically operated lid. The chamber sides were insulated with carbon impregnated foam (RFI Industries, VIC, Australia) to prevent RF-EME reflection. Small fans were implemented for external air circulation into the chamber through the base. RF-EME was generated by a Rohde and Schwarz SMC100A signal generator (Macquarie Park, NSW, Australia), connected to a signal amplifier. Chamber lid operation was controlled by a timed motor in order to raise or lower the lid every 12 h. Mice were housed in plastic cages with Perspex lids and plastic water bottles to ensure there was no metal, which interferes with RF-EME distribution. Cages were arranged radially around a central RF-EME emitting antenna, and oriented so that the water bottle furthest from the radiation source to minimize liquid interference. When mice were removed they were replaced with ‘phantoms’ composed of a 50 ml Falcon tube filled with 142 mM NaCl in deionized water to mimic blood. Sham exposed males were placed in the waveguide under identical conditions, however, the signal generator was turned off, thus receiving no exposure to RF-EME. All treatment groups were sacrificed at three time points; 1, 3 and 5 weeks of exposure and compared to an untreated control population of mice that were not placed inside the chamber. Mice were weighed weekly throughout the treatment regime (EME or sham exposed) during the time the waveguide lid was open. The weights were recorded after mice were individually placed in a tared container on top of the weigh tray of an electronic balance.

The SAR delivered to the mice was calibrated using a NARDA NBM 520 electric field meter with an EF1891 probe to measure electric fields in the empty irradiation system. Radial electric field measurements were made as a function of distance from the vertical aerial mounted in the center of the system after the antenna length was adjusted to maximize power supplied to the system at a frequency of 905 MHz. For 1 W input to the aerial a maximum electric field of 94 V/m was measured 16 cm from the center, whereas in their slightly larger setup, Puranen *et al*.^23^ measured a maximum electric field of 80 V/m at 15 cm from the center. The variation of E field with radial distance and the maximum electric fields in the two setups were found to be similar for the same power input.

The SAR (W kg-1) is related to the electric field, E, in a sample of conductivity σ (S m-1), and density ρ (kg m-3) by

1 $${\rm{SAR}}=\sigma |{\rm{E}}|\,2/{\rm{\rho }}\,({\rm{Wkg}}-1)$$

where E is the root-mean-square local electric field strength in V m-1. Puranen *et al*. (2009) measured a SAR of 0.11 W/m for the above 1 W input to the aerial. During our irradiations the input RF power was 20 W, corresponding to an average SAR of 2.2 W/kg since the geometry of our irradiation system is very similar to that of Puranen *et al*.^23^.

### Assessment of testis sections

Upon dissection, testes were fixed in Bouin’s solution, sectioned, dewaxed and rehydrated using standard protocols^47^. One section from each testis was stained with hematoxylin and eosin to investigate testis morphology, while the remainder were prepared for immunohistochemistry as previously described^48^. Antigen retrieval was performed by microwaving slides in 50 mM Tris (pH 10.5) for 9 min. Tissue sections were blocked (3% bovine serum albumin (BSA)-PBST, 10% goat serum) for 1 h at room temperature, washed in PBS for 5 min and labeled with appropriate pairs of primary (either anti-phospho-γH2AX (2 µg/ml) or anti-4-hydroxynonenal (1/300) antibodies in 1% BSA-PBST overnight at 4 °C) and AlexaFluor-conjugated secondary antibodies (1 h at 37 °C). After washing in PBS, sections were counterstained with DAPI (0.5 µg/ml), and viewed using fluorescence microscopy. Mean pixel intensity analysis was conducted on images using ImageJ version 1.48 V (NIH, USA). Pixel intensity determination was performed only on the seminiferous tubules, with surrounding interstitial tissue isolated from this analysis. For γH2AX, meiotic germ cells were excluded from the analysis due naturally occurring high levels of double strand breaks in these cells^50^.

### Preparation of spermatozoa

Epididymides were dissected immediately after euthanasia and mature spermatozoa were collected from the caudal segment by retrograde perfusion before being resuspended in 1 ml of modified Biggers, Whiting, Whittingham media (BWW)^49^. Objective sperm motility was assessed by computer assisted sperm analysis (IVOS, Hamilton Thorne, Danvers, MA, USA) as previously described^50^, and sperm vitality was determined via eosin exclusion.

### Determination of ROS production in spermatozoa

Spermatozoa were assessed for ROS generation via flow cytometry with the mitochondrial superoxide probe MitoSOX Red (MSR) or cytosolic superoxide probe dihydroethidium (DHE) in conjunction with Sytox Green (SYG) vitality stain as previously described^51^.

### Sperm chromatin dispersion (Halo) assay

Spermatozoa were snap frozen in liquid nitrogen and stored at −80 °C prior to analysis. Spermatozoa were defrosted and mixed with 1% low melting point agarose at 37 °C and applied to Superfrost slides (Thermo Fisher Scientific) pre-coated with 0.65% agarose. The slides were sealed with a coverslip and placed at 4 °C to solidify for 5 min. After removing the coverslips, the slides were treated with 0.08 N HCl for 7 min in foil, followed by Halo solution 1 (pH 7.5; 0.4 M Tris, 1% SDS, 50 mM EDTA, 0.8 M DTT) for 10 min and Halo solution 2 (pH 7.5; 0.4 M Tris, 1% SDS, 2 M NaCl) for 5 min at room temperature to lyse the cells, relax and neutralize the DNA. Next, slides were exposed to Tris-boric acid-EDTA buffer (pH 7.5; 0.1 M tris, 0.09 M boric acid, 0.002 M EDTA) for 2 min, then washed in ethanol (70%, 90% then 100%) for 2 min each to dehydrate the slides. After air drying, slides were counterstained with DAPI (0.5 µg/ml) for 10 min at room temperature, rinsed in PBS and mounted.

### Alkaline comet assay

The alkaline comet assay was performed as described previously^52^. DNA damage was analysed using Comet Assay IV software (Perceptive Instruments, Suffolk, UK). Hydrogen peroxide treatment (500 µM, 5 min at room temperature) was utilized as a positive control. To compare sperm DNA damage between treatments, percentage tail DNA values of each cell in the treated samples were normalized to that of the average percentage tail DNA of the respective untreated control for each time point. The control itself taking on the value of 1. The normalized data for each sample then contributed to a biological replicate. The average of these replicates are then graphed. The normalization process is required to minimize the noise generated by the small fluctuations in tail intensity between independent runs and days.

### Oxidative DNA damage assay

Oxidative DNA damage was assessed by suspending 2 × 10^6^ spermatozoa in Oxidative DNA/RNA damage antibody (Thermo Fisher Scientific) diluted 1/40 in PBST overnight at 4 °C. Cells were then centrifuged for 5 min at 450 × g and washed in PBS before incubation in AlexaFluor-488 goat α rabbit secondary (Abcam, Massachusetts, US) diluted 1/400 in PBST for 1 h at 37 °C. Finally, cells were again washed and resuspended in PBS for counting and imaging via fluorescence microscopy.

### Sperm functional assays and *in vitro* fertilization

Cauda epididymal spermatozoa were assessed for their ability to undergo capacitation-associated tyrosine phosphorylation, a calcium ionophore (A23187) induced acrosome reaction and bind zona pellucidae as previously described^53,54^. Alternatively, 2 × 10^5^ capacitated spermatozoa were inseminated into a droplet of oocytes recovered from superovulated female C57BL/6 mice^55^. The gametes were co-incubated for 4 h at 37 °C prior to the oocytes being assessed for fertilization (i.e. extrusion of second polar body and/or pronucleus formation). Zygotes were cultured in HTF medium overnight and transferred into G1 PLUS culture medium (Vitrolife, Stockholm, Sweden) on the morning of day 2 followed by an additional media change into G2 PLUS medium (Vitrolife) on Day 4^55^. The percentage of fertilized oocytes as well as embryos that had reached blastocyst stage by the morning of day 5 was calculated.

### Study design

Twenty adult male mice were randomly assigned to three treatment groups (untreated control, sham exposure control, RF-EME exposed), determined by the number of mice that could fit in the waveguide (10 cages, 2 mice per cage). Six mice were randomly selected for the 7 and 21 day intervals, while eight mice were selected for the 35 day interval. After each interval the mice were phenotyped for male fertility. For the purpose of this study, we ran the experiment twice to generate sufficient numbers of biological replicates for certain assays, e.g. MitoSOX, where we used 11 replicates. Each of the two treatment cycles consisted of 6 males treated for 1 week, 6 males treated for 3 weeks and 8 males treated for 5 weeks. The individual number of replicates for each assay can be found within the figures and is also shown in Table 1, below. As 20 mice were utilized for end point assays over the period of 5 weeks, the reduction in the number of replicates for body weight measurements decreases with the use of these individuals at the 1 and 3 week time points accordingly.

**Table 1:** Number of replicates used for each assay.

Assay/measurement	Untreated control	1 week		3 weeks		5 weeks	
		Sham	EME	Sham	EME	Sham	EME
Body weight	NA	20	20	14	14	8	8
Testis histology	3	3	3	3	3	3	3
Testis staining: γH2AX	3	3	3	3	3	3	3
Testis staining: 4-hydroxynonenal	3	3	3	3	3	3	3
Sperm vitality	5	6	6	5	6	8	8
Sperm motility, progressive and rapid	5	5	6	5	6	8	8
Dihydroethidium staining	4	6	9	5	8	8	8
MitoSOX red staining	6	6	12	5	11	11	11
DNA fragmentation	5	6	6	5	6	8	8
Comet tail intensity	3	3	3	3	3	3	3
8OHdG	3	4	4	4	5	5	5
Tyrosine phosphorylation	3	NA	NA	NA	NA	3	3
Acrosome reaction	3	NA	NA	NA	NA	3	3
Sperm-zona pellucida adhesion	3	NA	NA	NA	NA	3	3
Fertilization	7	NA	NA	NA	NA	3	5
Blastocyst development	3	NA	NA	NA	NA	3	3

### Statistical analysis

Samples from each animal were considered as a single biological replicate. Experimental data was analyzed using JMP version 11 software (SAS Institute Inc., Cary, NC). Normality of datasets was assessed with the Shapiro-Wilks test (α = 0.05). A one-way ANOVA was used to compare normally distributed treatments, with a post-hoc Tukey’s honest significant difference test (α = 0.05). For data not normally distributed, the Wilcoxon test was used (α = 0.05), with a post-hoc Dunn’s test. Error bars represent standard error values around the mean.

### Ethics statement

All experimental protocols were approved by the University of Newcastle (UON) Animal Care and Ethics Committee (Ethics Number 2014–447) and were performed in accordance with national and international guidelines, including the NSW Animal Research Act 1998, NSW Animal Research Regulation 2010 and the Australian Code for the Care and Use of Animals for Scientific Purposes 8th Ed.

## Data Availability

All data generated or analyzed during this study are included in this published article.
